# Development of Attention to Faces during the First 3 Years: Influences of Stimulus Type

**DOI:** 10.3389/fpsyg.2017.01976

**Published:** 2017-11-17

**Authors:** Klaus Libertus, Rebecca J. Landa, Joshua L. Haworth

**Affiliations:** ^1^Learning Research and Development Center, University of Pittsburgh, Pittsburgh, PA, United States; ^2^Center for Autism and Related Disorders, Kennedy Krieger Institute, Baltimore, MD, United States; ^3^Psychiatry and Behavioral Sciences, Johns Hopkins University School of Medicine, Baltimore, MD, United States; ^4^Science & Learning Center, Whittier College, Whittier, CA, United States

**Keywords:** face preference, attention, eye tracking

## Abstract

The development of attention toward faces was explored during the first 3 years of life in 54 children aged between 3 and 36 months. In contrast to previous research, attention to faces was assessed using both static images and a dynamic video sequence in the same participants. Separate analyses at each age and exploratory longitudinal analyses indicate a preference for faces during the first year, followed by a decline during the second year. These results suggest that attention to faces does not follow a linear increasing pattern over development, and that social attention patterns are influenced by stimulus characteristics.

## Introduction

To access the social and communicative information present in faces, young children need to detect and attend to other people. Attention to people's faces in particular has been examined extensively during the first months of life (e.g., Maurer, 1985; Johnson et al., 1991) but its developmental trajectory beyond infancy has received less attention. The current study fills this gap by using eye tracking to assess how attention to faces in the presence of distracting non-social stimuli changes during the first 3 years of life. To examine influences of stimulus type, we compare the development of a preference for faces between static photographs and dynamic video sequences.

### The development of a preference for faces

Across a range of species—including human infants—heightened interest in faces compared to other, non-social stimuli has been observed during the newborn period (e.g., Goren et al., 1975; Valenza et al., 1996; Sugita, 2008; Rosa Salva et al., 2011). This early attention to faces may be driven by invariant perceptual features characteristic of all faces (such as, up-down asymmetry, for review see Simion et al., 2007). However, infants' preference for faces does not follow a linear increasing trajectory but declines around the second month, only to re-emerge by 5 months of age (e.g., Johnson et al., 1991; Turati et al., 2005; Chien, 2011; Ichikawa et al., 2013). This non-linear developmental trajectory has sparked several studies on infants' attention to faces. Tables 1, 2 provide an overview of studies that have examined infants' interest in faces (in the absence of distractors) or preferential looking at faces (in the presence of one or more distractors, referred to “face preference” in the following). All studies reported in Tables 1, 2 have used looking duration as dependent variable. Studies using a manual response such as, a head turn (Goren et al., 1975; Johnson et al., 1991) are not included. Table 1 summarizes findings from studies using static images as stimuli, whereas Table 2 summarizes studies using animated stimuli (such as, video).

**Table 1:** Overview of studies on face preference using static images.

**Age**	**Study**	**Stimulus type**	**Distractors**	**Face preference?**
0	Fantz, 1963	Drawing	None	Yes
0	Valenza et al., 1996	Pattern	One	Yes
0.5	Keller and Boigs, 1991	Drawing	None	No
1	Maurer and Barrera, 1981	Drawing	None	No
1.5	Keller and Boigs, 1991	Drawing	None	No
2	Maurer and Barrera, 1981	Drawing	None	Yes
2	Ichikawa et al., 2013	Pattern	One	No
2.5	Keller and Boigs, 1991	Drawing	None	No
3	Ichikawa et al., 2013	Pattern	One	No
3	Libertus and Needham, 2011	Realistic	One	No
3	Libertus and Needham, 2014b	Realistic	One	No
3	Turati et al., 2005	Realistic	One^*^	Yes
3	Di Giorgio et al., 2012	Realistic	Multiple	No
3.5	Keller and Boigs, 1991	Drawing	None	Yes
3–5.5	Chien, 2011	Realistic	One^*^	Yes
4.5	Keller and Boigs, 1991	Drawing	None	Yes
5.5	Keller and Boigs, 1991	Drawing	None	Yes
4–8	DeNicola et al., 2013	Realistic	One	Yes
5	Libertus and Needham, 2011	Realistic	One	Yes
5–11	Libertus and Needham, 2014b	Realistic	One	Yes
6	Gliga et al., 2009	Realistic	Five	Yes
6	Di Giorgio et al., 2012	Realistic	Multiple	Yes
6	Gluckman and Johnson, 2013	Realistic	Five	Yes
6	Schietecatte et al., 2012	Realistic	Multiple	Yes
Adult	Libertus and Needham, 2014b	Realistic	One	No
Adult	Di Giorgio et al., 2012	Realistic	Multiple	Yes
Adult	Chien, 2011	Realistic	One^*^	Yes

*Age is reported in months. All results are based on looking duration analyses. Studies without distractor used sequential presentation protocols*.

* *Distractor was an inverted face*.

**Table 2:** Overview of studies on face preference using dynamic stimuli.

**Age**	**Study**	**Stimulus type**	**Distractors**	**Face preference?**
2	Ichikawa et al., 2013	Pattern	One	Yes
3	Ichikawa et al., 2013	Pattern	One	Yes
3	Frank et al., 2009	Cartoon	Multiple	No
6	Frank et al., 2009	Cartoon	Multiple	Yes
6	Schietecatte et al., 2012	Realistic	Four	Yes
9	Frank et al., 2009	Cartoon	Multiple	Yes
3–30	Frank et al., 2012	Realistic	None	Yes, increasing with age
3–30	Frank et al., 2012	Realistic	Multiple	Yes, declining with age
Adult	Frank et al., 2009	Cartoon	Multiple	Yes

*Age is reported in months. All results are based on looking duration analyses*.

### Face preference using static stimuli

As can be seen in Table 1, a preference for faces or face-like images is evident in the newborn. This preference has also been found in classic studies using a head turn procedure (Goren et al., 1975; Johnson et al., 1991). However, this initial preference for faces seems to decline quickly and the majority of studies report no preference for faces between 1 and 3 months of age. Notable exceptions to this pattern are Maurer and Barrera (1981) who report a preference for faces in 2-month-old infants using a longer stimulus presentation (40 s or more), and Turati et al. (2005) who report a preference for natural over unnatural (scrambled) faces in 3-month-old infants. However, it should be noted that issues related to assessing preferences in very young infants (i.e., increased variability due to infant state) may mask a face preference between 1 and 3 months of age. After 3.5 months of age, previous studies consistently report a preference for faces that is present until at least 11 months of age (Libertus and Needham, 2014b). Unfortunately, no prior studies have examined the development of a preference for faces beyond the first year in the context of static images, but some studies have provided data on adult comparison groups. Out of three previous studies, only one failed to report a preference for faces in adults (Libertus and Needham, 2014b). Together, these results show that static images of a face attract attention in newborns and again in infants after 3.5 months of age. Between 1 and 4 months a preference for faces seems absent, but factors such as, stimulus presentation duration or presence and type of distractors can strongly influence infants' attention to faces.

### Face preferences using dynamic stimuli

Fewer studies have examined a preference for faces during infancy using dynamic or animated stimuli (see Table 2). In contrast to the pattern observed with static images, a preference for faces seems present from 2 to 30 months when dynamic stimuli are used (e.g., Schietecatte et al., 2012; Ichikawa et al., 2013). One cross-sectional study with data from 129 children between the ages of 3–30 months reported a significant preference for faces at all ages when only a single face was displayed (Frank et al., 2012). However, attention to faces dropped below 50% of the total looking duration after the first year when competing visual stimulation was present. In particular, competing social stimulation such as, seeing the actor's hands move increasingly attracted attention after the first year. A preference for faces has also been reported in adult participants during observation of animated video sequences (Frank et al., 2009). Together, these findings suggest that a preference for faces seems present earlier when observing dynamic stimuli rather than static images.

Despite the longstanding interest in infants' attention toward faces, only two prior studies have examined the impact of stimulus type (static vs. dynamic) on attention and these two studies report findings that vary with age. One study reported an effect of stimulus type at 3 months (Ichikawa et al., 2013), with no face preference using static stimuli but a significant preference using dynamic stimuli. In contrast, the other study reported no effect of stimulus type at 6 months of age (Schietecatte et al., 2012), with a significant face preference in both static and dynamic conditions. Consequently, open questions remain regarding the development of a preference for faces over the first years of life and the impact of stimulus type (static vs. dynamic) on this preference.

### The current study

The current study uses separate analyses within each age-group tested to describe the developmental trajectory of attention toward faces in both static and dynamic contexts between 3 and 36 months of age. In addition, exploratory longitudinal analyses with a sub-set of participants examine how age and stimulus type interact during development. Based on previous findings, we predict a strong and increasing preference for faces from 3 to 10 months of age for both static and dynamic stimuli. However, we also hypothesize that attention to faces will decline in the static context following the first year of life (see Table 1). In contrast, we predict a preference for faces to be maintained in the dynamic context with increasing age (see Table 2). By comparing static and dynamic stimuli across a wide range of ages, the current study describes the developmental changes in social attention during the first 3 years and addresses questions that rain open in the literature to date.

## Methods

### Participants

A total of 54 children participated in this experiment as part of an ongoing longitudinal study. All participants came from middle- to upper-class backgrounds with an average Socioeconomic Status (SES) score of 54.99 (Hollingshead, 1975). Based on parent report, 45 identified as white, five as African American, and four as more than one race. Assessments were conducted at eight separate ages (at 3, 6, 10, 14, 18, 24, 30, and 36 months). However, participants were not required to begin the study at 3 months of age or to complete more than one visit to the study. Rather, visits were considered separate studies and participants were recruited separately for each visit (and re-consented at each visit). As a consequence, 15 participants (28% of sample) completed only a single visit, 7 (13%) completed two visits, and 32 (59%) completed three or more visits (across all participants: *M* = 3.46 visits, *SD* = 2.13, range 1–8 visits). This results in a mixture of single-visit and longitudinal data for the current study (see Table 3). This structure of the data limits our analytical approach as discussed in the Analyses section below. Together, the 54 participants provided data on 187 eye tracking sessions that were analyzed.

**Table 3:** Sample characteristics.

**Group**	***N***	**Number of females**	**Age (months)**	**Longitudinal sample**
3-month-olds	14	8	3.71 (0.63)	6
6-month-olds	31	15	6.73 (0.62)	13
10-month-olds	33	21	10.58 (0.47)	16
14-month-olds	31	20	14.75 (0.75)	16
18-month-olds	25	16	18.78 (0.87)	14
24-month-olds	22	15	24.31 (0.74)	9
30-month-olds	19	11	30.39 (0.90)	–
36-month-olds	12	6	37.32 (0.78)	–

*Longitudinal sample includes participants completing three consecutive visits starting at the listed age. Values in parenthesis are SD*.

### Procedure

The Institutional Review Board of the Johns Hopkins School of Medicine approved all methods in this study and informed written consent was obtained by a parent of each child prior to their participation in this study. Testing was conducted in a small, dimly lit room where children were seated in a stable high chair or on their parent's lap at a distance of about 60 cm from a Tobii X120 remote cornea-reflection eye-tracker with 120 Hz sampling rate. Stimuli were presented on a 22″ screen (43.60 × 28.1 degrees of visual angle) at a resolution of 1,680 × 1,050 pixel. A nine-point calibration procedure was performed with each participant prior to data collection and repeated until at least five usable calibration points were obtained.

### Tasks

All participants completed the same eye tracking session that included two independent tasks to assess their preference for faces: a static preference task and a dynamic preference task. Presentation of stimuli was randomized across participants. Usable data was defined as on-screen looking durations >25% of the trial duration. Trials that failed to meet this requirement were removed from analyses. In three instances, no usable data was obtained during the dynamic preference task. The total number of eye tracking sessions in the final analyses was 187 for the static task and 184 for the dynamic task.

#### Static preference task

**Figure 1:**
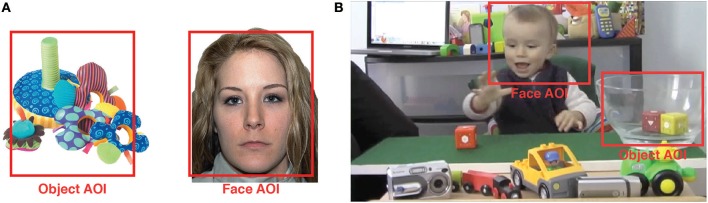
Examples of the stimuli used to assess attention to faces using **(A)** static images, and **(B)** dynamic video. Red squares show Areas of Interest (AOI) used for analyses. Note that **(A,B)** in this figure are not displayed on the same scale. The face in a is taken from the NimStim set (Tottenham et al., 2009), the image of the child in b is used with written permission by both parents of the child.

This task has been used in five previous studies investigating infants' preference for faces over objects and consists of eight face-toy pairs that are presented for a fixed duration of 10 s each (Libertus and Needham, 2011, 2014a,b; DeNicola et al., 2013; Libertus and Landa, 2014). Face-toy pairs were constructed by placing photographic images of a face and a toy side-by-side on a white background (Figure 1A). Faces were selected from the NimStim face database (Tottenham et al., 2009) and displayed a neutral facial expression. Faces and toys were 3.8–6.4 cm apart and similar in size and overall luminance (DeNicola et al., 2013). To maximize ecological validity, faces, and toys were not equated for visual saliency.

#### Dynamic preference task

This task consisted of a 21-s video showing a toddler seated at a table with toys visible around him, and a clear bowl at the right (Figure 1B). An adult hand reaching in from the left sequentially placed four colorful blocks in front of the toddler who then grasped each block, one at a time, and dropped it into the bowl (see Supplemental Video 1). Upon being dropped into the bowl, blocks visibly bounced inside the bowl and produced impact sounds. Movement cues were present in both the child's face and inside the target bowl once blocks were dropped (bouncing blocks)—allowing for a direct comparison of participant's attention to the actor's face vs. the target bowl. During the actions, the toddler in the video smiled, vocalized, clapped, and visually examined the blocks. To increase ecological validity, the original audio track of the video was played. This included the impact sound of the blocks and voices of adults talking quietly in German and English. However, no adults were visible in the video and the voices only served to eliciting general attention toward the screen. The video sequence was shown twice to each participant.

### Analyses

#### Data processing

Due to the large age range of the participants tested here (ranging from 2.98 to 38.66 months of age), an assumption-free approach to analyzing eye gaze was used (see also Libertus and Needham, 2011, 2014b). Instead of defining fixations using an arbitrary filter algorithm, raw eye-gaze was used as a time-varying signal recorded at 120 Hz. Saccades were defined individually for each subject based on their specific gaze velocity profile as points where eye velocity exceeded 1 *SD* of their mean eye velocity and were removed from the data prior to analysis. Blinks and instances where tracking validity was low (<4 on a four-point scale) were treated as missing data and removed prior to analysis.

#### Areas of interest

Rectangular Areas of Interest (AOIs) were drawn around the faces and objects in the static context (each AOI sized 600 × 700 pixel), or around the actor's face (360 × 290 pixel) and around the target bowl where toys were dropped (360 × 260 pixel) in the dynamic context (see Figure 1). The target bowl was chosen as contrast for the face area in the dynamic condition because of its similar size, the shared presence of local motion and sounds, and its pragmatic importance during the video sequence (i.e., it is the goal of the child's actions). Looks outside AOIs were removed from analysis (%Face + %Object = 100%) and a single face-preference score was calculated separately for the static and the dynamic context (FP = %Face – %Object). Using this score, positive values indicate a face preference, negative values an object preference, and 0 indicates no preference (Libertus and Needham, 2011).

### Statistical analyses

**Figure 2:**
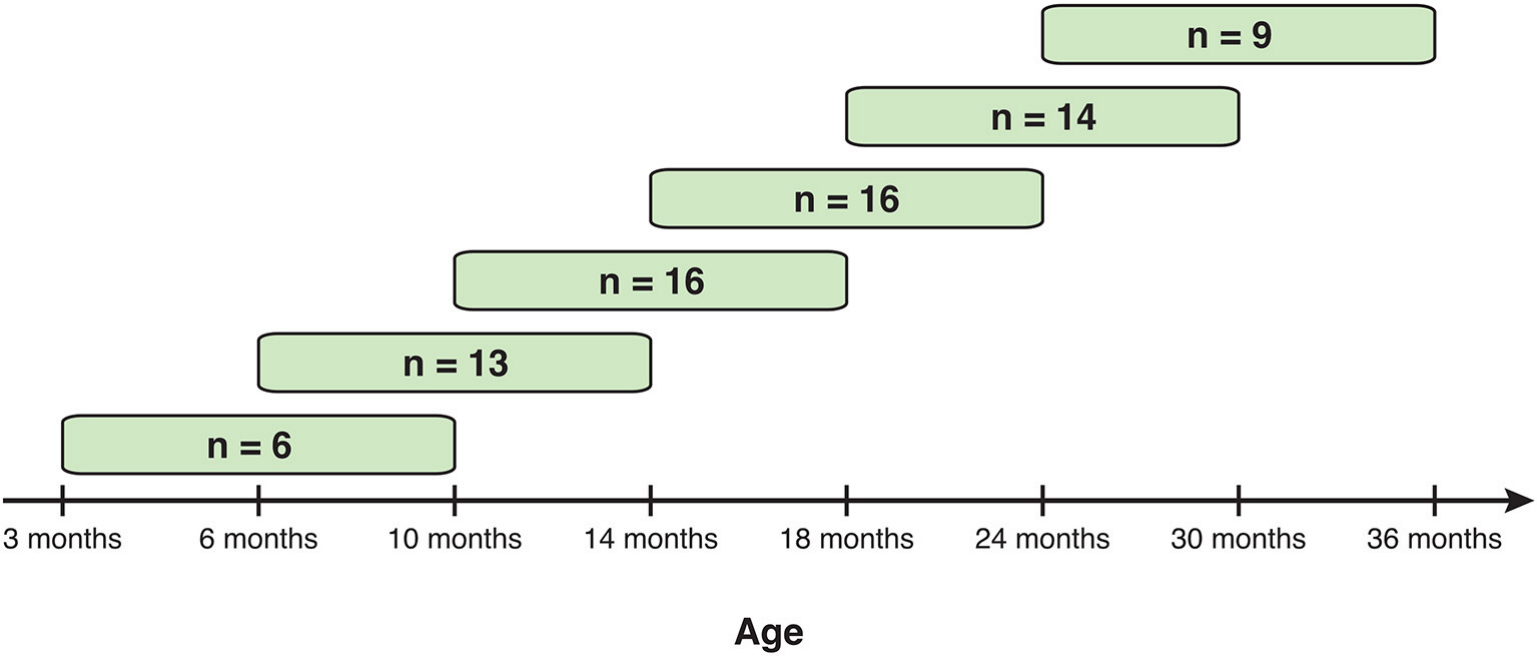
Sample size in each of six sliding age-windows used in longitudinal analyses. Each time-window overlaps with the previous and following window and may contain some of the same participants.

Three complementary analyses were conducted. First, face preferences were examined separately at each age (see Table 3 for ages and groups sizes) and for each stimulus type. This first analysis establishes whether a face preference is present or absent at each of the ages observed here. However, this first approach does *not* compare between age groups as some participants provided data at more than one age (violating assumptions of independence, see longitudinal analyses) and does not compare face-preference scores between stimulus types. Second, the effects of age and stimulus type on face preference were examined using longitudinal analyses of six sliding age-windows. Only participants who completed three or more *consecutive* visits (see Figure 2 for group sizes) were included and this approach allows comparisons across age without imputing missing data. Third, the longitudinal analyses were repeated over the entire age-range by imputing missing observations.

#### 1) Separate age group analyses

The presence of a preference for faces was examined using single-sample *t*-tests (two-tailed, comparing face-preference scores to 0; note that log transformed data is compared to ln(100) instead of 0) for each age assessed here. This approach is similar to the analyses used in previous studies on face preference (Libertus and Needham, 2014b). Bonferroni correction for two comparisons with data from the same participants (static context and dynamic context) were applied to these analyses (i.e., original *p*-values were multiplied by 2 in Table 4).

**Table 4:** Statistics comparing attention to faces by age and context.

	**Static context**		**Dynamic context**	
**Age**	**Statistic**	**Mean**	**Statistic**	**Mean**
3-month-olds	*t*(13) = 2.081, *p* = 0.116; *d* = 1.15	21.68 (38.98)	***t***(12) = **4.800**, ***p*** < **0.001;** ***d*** = **2.77**	34.84 (26.17)
6-month-olds	***t***(30) = **8.166**, ***p*** < **0.001;** ***d*** = **2.98**	40.10 (27.34)	***t***(30) = **5.987**, ***p*** < **0.001;** ***d*** = **2.19**	30.84 (28.68)
10-month-olds	***t***(32) = **5.098**, ***p*** < **0.001;** ***d*** = **1.80**	25.59 (28.83)	***t***(30) = **4.314**, ***p*** < **0.001;** ***d*** = **1.58**	20.98 (27.09)
14-month-olds	***t***(30) = **3.146**, ***p*** < **0.001;** ***d*** = **1.15**	14.70 (26.02)	*t*(30) = 0.739, *p* = 0.931; *d* = 0.27	2.83 (21.33)
18-month-olds	*t*(24) = −1.432, *p* = 0.330; *d* = 0.58	−7.65 (26.73)	*t*(24) = 0.494, *p* > 0.99; *d* = 0.20	4.53 (23.90)
24-month-olds	*t*(21) = −0.278, *p* > 0.99; *d* = 0.12	1.22 (24.60)	*t*(21) = 2.304, *p* = 0.063; *d* = 1.01	10.24 (20.84)
30-month-olds	*t*(18) = −0.011, *p* > 0.99; *d* = 0.01	−0.08 (30.51)	*t*(18) = 0.266, *p* > 0.99; *d* = 0.13	0.92 (15.11)
36-month-olds	*t*(11) = 0.594, *p* > 0.99; *d* = 0.36	4.81 (28.06)	*t*(11) = 1.644, *p* > 0.257; *d* = 0.99	10.49 (22.09)

*Significant results are highlighted in bold. d = Cohen's d. Comparisons are Bonferroni corrected (for 2 comparisons), using p > 0.99 if correction resulted in a value >1.0*.

#### 2) Longitudinal analyses

Longitudinal analyses were conducted with a subset of participants who completed three *consecutive* assessments, using repeated measures Analysis of Variance (ANOVA) with Age and stimulus Context (static vs. dynamic) as repeated factors. To make use of all available data and to cover all ages available in our sample, a sliding-age-window approach was selected for these analyses (see Figure 2). The primary purpose of the longitudinal analyses was to confirm and extend the results of the within-age group analyses over time in the same children.

#### 3) Longitudinal analyses with imputed data

An alternative approach to analyzing our longitudinal data is to use data from all participants who completed at least three assessments (not required to be consecutive, *n* = 32) and to fill in any missing observations using multiple imputation. For these analyses, 10 imputations of the missing values were calculated using SPSS. An Age (8) by Stimulus (2) repeated measures ANOVA was then calculated for each imputation, and statistics were averaged across the 10 imputed data sets. Due to violations of sphericity in the imputed data, multivariate tests using Wilks' Lambda are reported for this analysis.

## Results

### Preliminary analyses

All data were examined for violations of normality. Data for the dynamic task at 18 months (*p* = 0.013) and for the static task at 24 months (*p* = 0.023) showed departures from normality and were log-transformed. Transformed data did not violate normality. To examine potential influences of Gender, separate independent sample *t*-tests were performed at each age comparing boys' with girls' attention to faces in static and dynamic contexts. Results revealed differences in face preference between girls and boys at 3 months of age in the static context, *t*_(12)_ = 2.40, *p* = 0.033, *d* = 1.40 (*M*_*Female*_ = 40.23, *SD*_*Female*_ = 30.21; *M*_*Male*_ = −3.05, *SD*_*Male*_ = 37.28) and at 36 months in the dynamic context, *t*_(10)_ = 2.79, *p* = 0.019, *d* = 1.76 (*M*_*Female*_ = 24.46, *SD*_*Female*_ = 22.23; *M*_*Male*_ = −3.50, *SD*_*Male*_ = 10.50). In both case, female participants showed a stronger preference for faces than male participants. However, sample sizes were very small for these comparisons. No other effects of Gender were observed (*ps* > 0.066) and this factor was not considered in subsequent analyses.

#### 1) Separate age group analyses

**Figure 3:**
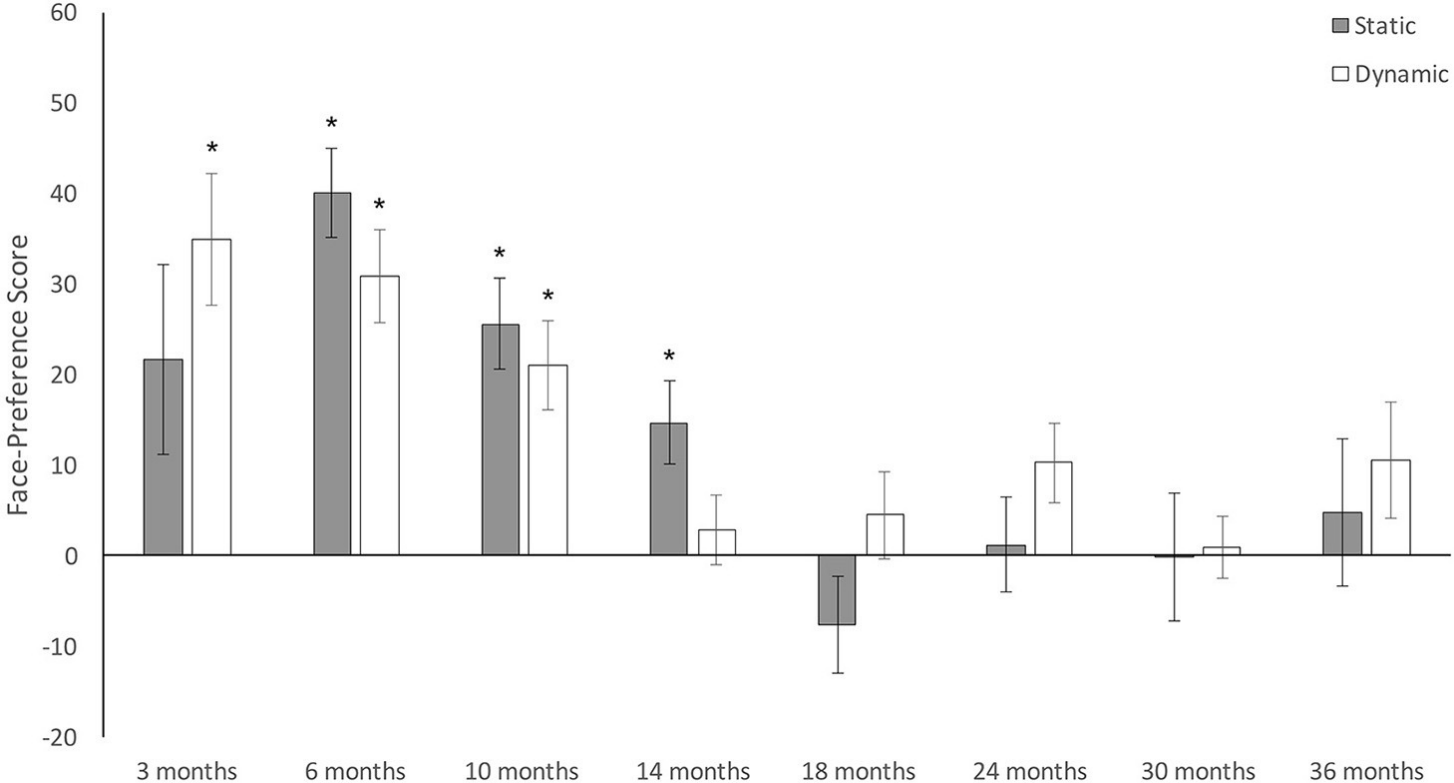
Results from separate analyses on face preference scores at each age and for each stimulus type. Error bars are SEM. ^*^p < 0.05.

Results for this set of analyses are summarized in Table 4 and Figure 3. Overall, results reveal very similar patterns for both static and dynamic stimuli across age. In the static context, infants showed a significant preference for a face over a toy distractor at 6, 10, and 14 months of age. No face preference was observed at 3, 18, 24, 30, or 36 months. Similarly, in the dynamic context, infants showed a significant preference for the actor's face over the target bowl at 3, 6, and 10 months of age. No face preference was observed at 14, 18, 24, 30, or 36 months (however, at 24 months results were marginal with *p* = 0.063). Thus, children show preferential attention to faces between 3 and 14 months of age. Further, at 6 and 10 months of age, this preference seems stimulus independent and was observed in both the static and dynamic context. In contrast, at 3 and 14 months, stimulus animation did influence face-preference scores. Together, these results confirm our first hypothesis of a strong and increasing preference for faces across contexts during the first year, and our second hypothesis of a decline in attention to faces during the second year in the static context. However, counter to our original predictions, attention to faces also declined during the second year when using dynamic stimuli.

#### 2) Longitudinal analyses with sliding age-windows

Longitudinal analyses were conducted with children who provided data on three *consecutive* assessments using six sliding age-windows (see Figure 2). Changes in attention to faces were examined using repeated measures ANOVAs with Age (3) and Stimulus (2) as within-subject factors. Main effects of Age were not followed by *post-hoc* comparisons as the preceding analyses already detailed age-related changed in attention to faces.

No significant effects were observed during age-window 1 (3–10 months), age-window 5 (18–30 months), or age-window 6 (24 to 36 months; all *ps* > 0.065). These results suggest that children's preference for faces is not affected by age or stimulus type at these ages. However, the small sample sizes (*n*_1_ = 6, *n*_5_ = 14, *n*_6_ = 9) for these windows may limit power to detect significant differences.

**Figure 4:**
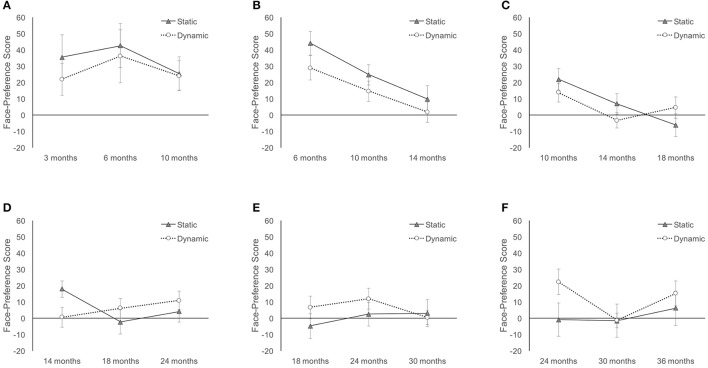
Longitudinal face-preference scores on sub-set of participants who participated across three consecutive age-points. Error bars are SEM. **(A)** 3–10 months, **(B)** 6–14 months, **(C)** 10–18 months, **(D)** 14–24 months, **(E)** 18–30 months, and **(F)** 24–36 months.

During age-window 2 (6–14 months), the ANOVA revealed a significant effect of Age, *F*_(2, 24)_ = 8.603, *p* = 0.002, $n_{p}^{2}$ = 0.418, and a significant effect of Stimulus, *F*_(1, 12)_ = 5.117, *p* = 0.043, $n_{p}^{2}$ = 0.299. However, the Age × Stimulus interaction failed to reach significance (*p* = 0.713). The effect of Stimulus seems driven by a stronger preference for faces in the static context across all three time points (see Figure 4B).

During age-window 3 (10–18 months), the ANOVA revealed a significant effect of Age, *F*_(2, 30)_ = 9.049, *p* = 0.001, $n_{p}^{2}$ = 0.376, but no effect of Stimulus (*p* = 0.553). The main effect of Age was qualified by a significant Age × Stimulus interaction, *F*_(2, 30)_ = 4.527, *p* = 0.019, $n_{p}^{2}$ = 0.232. While face-preference scores declined for static stimuli over this time period, scores stabilized for dynamic stimuli around 18 months of age (see Figure 4C).

During window 4 (14–24 months), the ANOVA revealed no main effects of Age (*p* = 0.152) or Stimulus (*p* = 0.912), but a significant Age × Stimulus interaction, *F*_(2, 30)_ = 5.144, *p* = 0.012, $n_{p}^{2}$ = 0.255. During this time period, face-preference scores increased for dynamic stimuli but declined for static stimuli (see Figure 4D). This pattern suggests a rebounding of attention to faces toward the end of the second year.

Together, these longitudinal analyses confirm our separate age analyses by showing a decline in face-preference scores over time. As is evident in Figures 4A–C, face-preference scores for static images peak at 6 months of age and then steadily decline until 18 months of age. Results are nearly identical for dynamic stimuli over this time period. However, interest in faces in a dynamic context rebounds after 14 months of age, while remaining essentially flat for static faces (Figures 4D–F).

#### 3) Longitudinal analyses with imputed data

**Figure 5:**
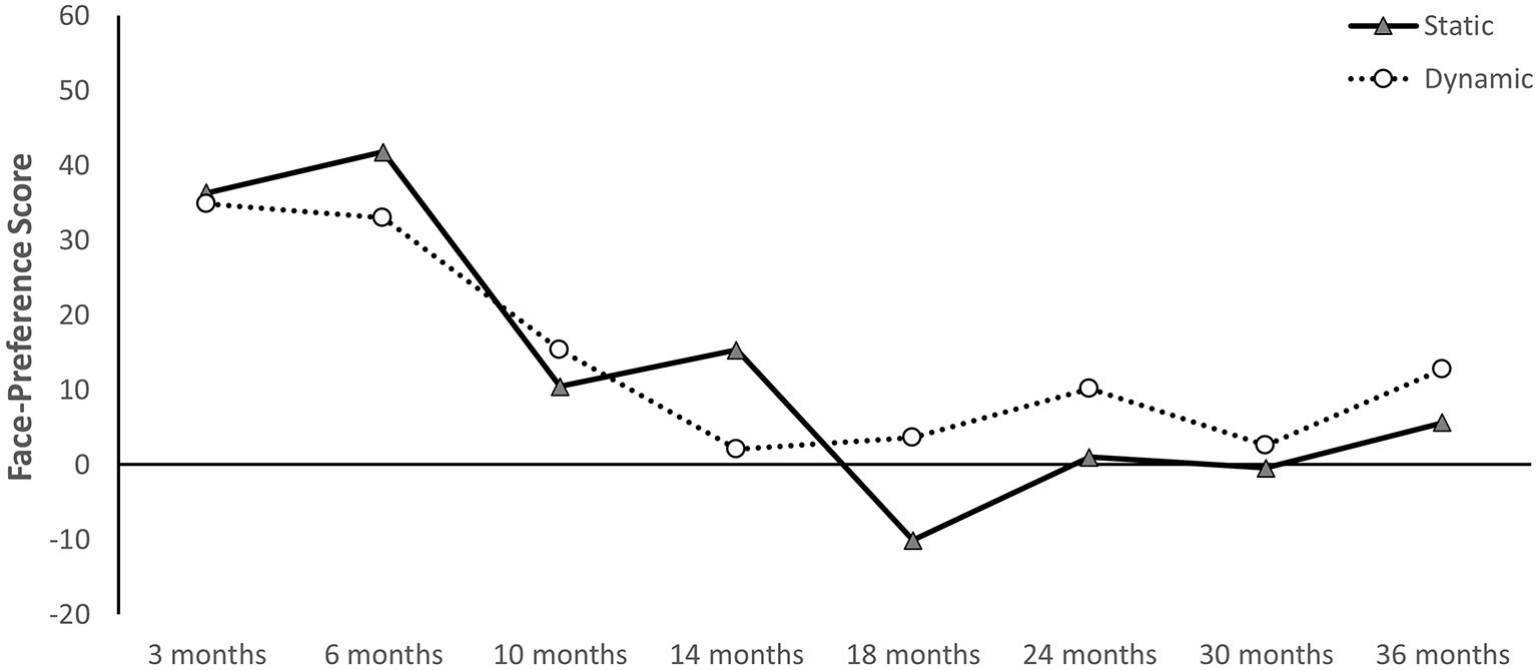
Longitudinal face-preference scores averaged across 10 data imputations.

Multiple imputation was used as alternative approach for the longitudinal analyses. All participants who completed three or more visits were included and data for missed visits was imputed using SPSS. Ten iterations were calculated and for each imputed dataset separate Age (8) by Stimulus (2) repeated-measures ANOVAs were calculated. The results reported here represent the average statistics across these 10 imputations. ANOVA revealed a significant main effect of Age, *F*_(7, 25)_ = 25.877, *p* < 0.001, $n_{p}^{2}$ = 0.87, but no main effect of Stimulus (*p* = 0.482). The main effect of Age was qualified by a significant Age × Stimulus interaction, *F*_(7, 25)_ = 4.838, *p* < 0.001, $n_{p}^{2}$ = 0.57. Average imputed means are displayed in Figure 5. Results using imputed data match the patterns observed without imputation (see Figure 4). A preference for faces peaks in early infancy and then declines until 18 months of age. Initially, face preference seems stronger when viewing static images, but starting around 18 months of age a stronger face preference is present when dynamic stimuli are used (Figure 5).

## Discussion

The present study investigated the developmental trajectory of attention toward faces in both static and dynamic contexts over the first 3 years of life. Two main findings can be derived from our results. First, preference for faces emerges during the first year and subsequently declines during the second year. Second, attention to faces is dependent on the stimulus type. In particular, static images seem to elicit a stronger preference for faces during the first year, whereas dynamic animations elicit a stronger preference during the second year of life. In fact, attention to faces shows signs of a rebound during the third year when viewing dynamic stimuli, suggesting a U-shaped developmental trajectory. In the following, we will discuss these results in the context of prior findings and their implications for future research.

### Attention to faces using static vs. dynamic stimuli

The majority of previous studies have used *either* static images *or* dynamic video displays to investigate children's attention to faces in the presence of distractors. To our knowledge, only two prior studies directly compared static and dynamic stimuli (Schietecatte et al., 2012; Ichikawa et al., 2013). The current findings confirm and expand these previous studies by examining the potential impact of stimulus type on attention to faces across the first 3 years of life.

Ichikawa et al. (2013) presented 2- to 3-month-old infants with face-like geometric patterns that either did or did not include internal movements. Their results revealed a preference for face-like patterns over linearly arranged top-heavy patterns, but only in the moving (i.e., dynamic) condition. Similarly, the current study reports a significant preference for faces in 3-month-old infants but only when using dynamic stimuli. Static images fail to elicit a significant face preference at this age, as has also been reported by previous studies using the same stimuli (Libertus and Needham, 2011, 2014b). In contrast, at 6 months of age, face-preference scores seem more robust. Schietecatte et al. (2012) explored 6-month-olds' attention to faces in static and dynamic contexts. Their findings reveal no effect of context with a significant preference for faces in both contexts. The current study confirms this pattern by reporting a significant preference for faces in both static and dynamic contexts at 6 months of age.

The developmental trajectory of attention to faces in static and dynamic contexts beyond the first year remains largely unknown. Frank et al. (2012) examined attention to faces in children aged between 3 and 30 months using dynamic stimuli. Their results showed an increase in attention to faces over time when no distractors were present, but a decrease in the presence of distractors (such as, the hands of the actor). Using static stimuli, Libertus and Needham (2014b) reported a decline in attention to faces in the presence of distractors after 9 months of age and no clear preference in adult participants. The current study supports both these findings and suggests a decline in attention to faces after 6 months of age in both static and dynamic contexts with distractors present. At the same time, our results reveal that a preference for faces develops differently in static and dynamic contexts. In a static context, a reliable preference for faces over toys emerges around 6 months of age (see also Libertus and Needham, 2011, 2014b) and is followed by a subsequent decline with no clear preference after 14 months of age. In contrast, in the dynamic context, attention to faces is evident between 3 and 10 months of age, then disappears but shows signs of a rebound toward the end of the second year and during the third year.

In addition to the findings reported here, it is worth noting that preferences for particular stimuli observed in infancy are often not stable and are influenced by age as well as by external factors such as, the experienced environment (Liu et al., 2015; Tham et al., 2017). While it has been well established that faces attract children's attention, an attentional bias that is potentially already present in the unborn fetus (Reid et al., 2017), it remains unknown attention to faces changes across ages as a function of experience and maturation of attentional mechanisms.

### Attention to faces in children at risk for ASD

The current study examined attention to faces in typically developing children but may have implications for children at high risk for Autism Spectrum Disorders (ASD). Specifically, our findings show that social attention is dependent on stimulus characteristics and this should be considered when examining social attention in children at risk for ASD. Reduced social attention has been reported in children later diagnosed with ASD (for review see Sasson, 2006). For example, retrospective observations using home video tapes suggest infants later diagnosed with ASD looked less at people (Osterling et al., 2002). Prospective studies using controlled in-lab procedures confirmed a reduced attentional bias toward faces, less attention to faces in social scenes, and less interest in complex social scenes in general in infants later diagnosed with ASD (e.g., Chawarska et al., 2010). The attenuation of social interest in ASD is particularly evident during observation of dynamic social scenes that include speech and directed gaze (Chawarska et al., 2013; Shic et al., 2014; Nele et al., 2015). This contrasts somewhat with the current findings, where dynamic stimuli elicited stronger social attention during the second year in typically developing children. These contrasting results may reflect differences between children with or without ASD. It is possible that dynamic, socially engaging stimuli as used here do *not* attract attention in children later diagnosed with ASD. Future research should explore this question by examining attention to faces in children later diagnosed with ASD.

## Limitations

Several limitations should be considered when interpreting the results of the current study. In particular, a large number of observations were missing from our dataset. Consequently, the patterns reported here need to be replicated in the future using larger longitudinal samples to further substantiate our understanding of how social attention develops over time. Another limitation is that we did not manipulate stimulus duration. Especially in the static context, presenting face-toy pairs for a longer period of time is likely to influence children's social attention (Maurer and Barrera, 1981). The effect of stimulus duration should be examined systematically in future research. The same hold for potential effects of stimulus type. In the static condition, adult faces were used, whereas in the dynamic condition the face of a child was shown. Previous work suggest that infants are more attracted by adult faces (Hernandez et al., 2009). This difference may have influenced results of the current study. Finally, due to the naturalistic nature of the stimuli used here, saliency was not carefully controlled between the face and the toy images. While our results argue against a systematic effect of visual saliency on our findings, it is possible that perceptual differences may have influenced the results reported here.

## Conclusions

The results of the current study advance our understanding of the development of social attention to faces in early childhood. Separate analyses with each age group reveal an initial increase in attention to faces during early infancy, followed by a decline during the second year. This pattern agrees with prior findings and was confirmed using both static stimuli and dynamic video sequences. A decline in attention to faces during the second year may be caused by increases in face processing skills and by a growing interest in other aspects of a social scene. At the same time, stimulus characteristics influence children's attention to faces. Face preferences are stronger for static images during the first year, but stronger for dynamic stimuli during the second and third year.

## Author contributions

KL and RL conceptualized and designed the study. KL and JH collected the data. KL analyzed the data and results were interpreted and examined by all authors. KL wrote the first draft of the manuscript. All authors edited and revised subsequent drafts of the manuscript. All authors agreed on the final version of the manuscript.

### Conflict of interest statement

The authors declare that the research was conducted in the absence of any commercial or financial relationships that could be construed as a potential conflict of interest.

## Supplementary material

Supplemental Video 1 Stimulus used for dynamic face preference task. The video shows a 15-month-old child placing blocks into a bowl. Dialogue of adults speaking in English and German could be heard in the background, but speakers were not visible in the video. Presence of the voices may have heightened infants' overall interest in faces during this task.
